# Clinical course, treatment and visual outcome of an outbreak of *Burkholderia contaminans* endophthalmitis following cataract surgery

**DOI:** 10.1186/s12348-021-00242-6

**Published:** 2021-04-19

**Authors:** Caroline Lind, Karina Olsen, Nina K. Angelsen, Einar A. Krefting, Kristian Fossen, Kirsten Gravningen, Eliza Depoorter, Peter Vandamme, Geir Bertelsen

**Affiliations:** 1 Department of Ophthalmology, University Hospital of North Norway, Tromsø, Norway; 2 Department of Microbiology and Infection Control, University Hospital of North Norway, Tromsø, Norway; 3 Department of Infection Prevention and Preparedness, Norwegian Institute of Public Health, Oslo, Norway; 4 Laboratory of Microbiology, Department of Biochemistry and Microbiology, Ghent University, Ghent, Belgium; 5 Department of Community Medicine, UiT – The Arctic University of Norway, Tromsø, Norway

**Keywords:** Endophthalmitis, Cataract surgery, Burkholderia contaminans, Outbreak

## Abstract

**Background:**

Postoperative endophthalmitis is a rare but dreaded complication of intraocular surgery and often results in severe visual impairment or blindness. The present study describes the clinical course, treatment and visual outcome of an outbreak of *Burkholderia contaminans* endophthalmitis following cataract surgery.

**Methods:**

Among 290 patients who underwent uneventful phacoemulsification cataract surgery at one outpatient clinic between January 4th and 28th 2019, 6 cases developed *Burkholderia contaminans* endophthalmitis. Clinical data were collected by retrospective review of patient records. Microbiological samples from vitreous aspirates, intraocular lenses (IOL) and lens capsules were cultured, and recA and draft whole genome sequences analysed.

**Results:**

The recA sequences of all *Burkholderia contaminans* isolates and the allelic profile of the isolates were identical. All cases had a similar clinical presentation with rapid development of endophthalmitis symptoms with variable time to onset. The mean time to admission was 34 days (12–112 days). All cases had a seemingly favourable response to intravitreal antibiotics. However, acute recurrences occurred after long time periods (12–71 days). The cases experienced between 0 and 3 recurrences. Due to persistent infection, the cases received between 5 and 15 treatments (mean 7.8) including IOL and lens capsule explantation in 5 of 6 cases. *Burkholderia contaminans* was detected in all explanted lens capsules. The final corrected distance visual acuity (CDVA, Snellen chart) was between 0.8 and 1.2 and all cases had final CDVA ≥0.8.

**Conclusions:**

A persistent and intensive treatment approach including total lens capsule and IOL explantation is recommended for *Burkholderia contaminans* endophthalmitis following cataract surgery and may lead to a favourable visual result.

## Background

Postoperative endophthalmitis is a rare but dreaded complication of intraocular surgery and often results in severe visual impairment or blindness. Due to improved surgical techniques and use of prophylactic intracameral antibiotics, the incidence of postoperative endophthalmitis following cataract surgery has decreased the last decades [1–3]. Endophthalmitis following cataract surgery is most commonly caused by Gram-positive bacteria, e.g. *Staphylococcus epidermidis*, originating from the patient’s skin flora [4]. The *Burkholderia cepacia* complex (BCC) is a group of Gram-negative bacteria composed of at least 22 closely related species, including *Burkholderia contaminans (B. contaminans)* [5]. BCC is ubiquitously found in nature, particularly in soil and water, [6] and resistance to antibiotics and antiseptics is common [7, 8].

Clinically, BCC is predominantly associated with chronic pulmonary infections in patients with cystic fibrosis, but may also cause infections in immunocompromised patients and patients with chronic granulomatous diseases [5]. Furthermore, BCC bacteria and in particular *B. contaminans* are commonly associated with contamination of pharmaceutical products such as nasal sprays, ultrasound gel, hand sanitizers, mouthwash and nebulization solutions [8, 9].

BCC endophthalmitis is uncommon and most of the reported cases occur after cataract surgery, although cases have also been reported after trauma, corneal transplantation, intravitreal injection and vitrectomy [10–15]. The visual outcomes following BCC endophthalmitis are often poor and many cases result in phthisis, enucleation or visual acuity (VA) of light perception [10, 12, 14–16].

In the present study, we report the clinical course, treatment and visual outcome in six confirmed cases of *B. contaminans* endophthalmitis following cataract surgery performed at one outpatient clinic.

## Methods

Clinical data were collected by retrospective review of patient records at the University Hospital of North Norway (UNN), Tromsø, Norway. From January 4th to 28th 2019, a total of 290 patients underwent uneventful phacoemulsification cataract surgery under topical anesthesia at one single private outpatient clinic. Among these, seven patients were referred to UNN with suspected postoperative endophthalmitis between January 26th and April 30th. Suspected endophthalmitis was defined according to the European Society of Cataract & Refractive Surgeons (ESCRS) guidelines [17], i.e. increasing intraocular inflammation based on the presence of reduced VA, increasing pain, red eye, increased anterior chamber flare, hypopyon and vitreous infiltration. All cataract surgeries were performed by one single experienced cataract surgeon. Two different single-piece intraocular lenses (IOL, i.e. B-Lens and BunnyLens AF) were implanted. UNN holds the only ophthalmology department in the region and all patients with suspected endophthalmitis are referred to this department. In addition, all patients who underwent cataract surgery at the private outpatient clinic during the time period of the outbreak were contacted by phone and invited to an eye examination if reporting any symptoms indicating endophthalmitis. The study was conducted according to the guidelines of the Declaration of Helsinki. All cases have given written consent to participate in the study and the study has been approved by the hospital data protection officer at UNN (No 02315). The Regional Committee for Medical and Health Research Ethics ruled that approval was not required for this study.

The date of endophthalmitis diagnosis was defined as the date of admission to the Department of Ophthalmology, UNN, with suspected postoperative endophthalmitis. Time to recurrence was defined as time from first intravitreal antibiotic injection during admission to following hospital admission with recurrent endophthalmitis symptoms. Number of treatments were counted and included intravitreal injections of antibiotics, IOL and lens capsule explantation and pars plana vitrectomy (PPV).

All cases underwent a clinical evaluation at presentation by an experienced ophthalmologist including slit lamp examination and ultrasonography. VA was measured using a Snellen chart. Based on the availability of a vitreoretinal surgeon, all cases with suspected endophthalmitis underwent either (i) an undiluted vitreous tap using one port and a vitrectomy cutter on the AMO WHITESTAR Signature phaco machine (Abbott Medical Optics Inc) or (ii) an undiluted vitreous tap combined with three port PPV (EVA vitrectomy system, DORC), and subsequent injection of antibiotics. In case of recurrent inflammation, all cases underwent PPV if not performed as the primary intervention. Finally, IOL explantation including lens capsule removal was performed in five cases.

Antibiotic and dexamethasone solutions for intravitreal injections were prepared according to ESCRS guidelines [17] and administered as 0.1 ml injections containing either ceftazidime 2 mg, gentamicin 0.2 mg, vancomycin 1 mg or dexamethasone 0.4 mg. Similarly, an intravitreal solution of 0,1 ml containing piperacillin/tazobactam 200/25 μg was used based on previous reports [16, 18].

Vitreous and anterior chamber samples were collected in separate sterile syringes. IOL and lens capsules were collected in sterile containers. All samples were sent to the Department of Microbiology and Infection Control, UNN, for microbiological analyses. The samples were cultured directly on standard, blood and chocolate agar plates (Oxoid, Thermo Fisher Scientific), and a selective *Burkholderia cepacia* medium (BCM) agar plate (Mast Group Ltd) for isolation of BCC at 37 °C for up to 5 days. If sufficient amount, the specimen was enriched on a 5% glucose broth incubated at 37 °C for up to 5 days. If there was no growth of bacteria at the directly cultured agar plates, the enrichment broths were recultured on blood and chocolate agar plates (Oxoid, Thermo Fisher Scientific) in addition to the BCM agar plate and incubated at 37 °C for up to 3 days. All isolates were identified as BCC with a score between 2.030 to 2.290 using Matrix Assisted Laser Desorption Ionization Time-Of-Flight mass spectrometry (MALDI-TOF MS) (Bruker Daltonics) with the MBT 7854 Compass Library, revision E. DNA of all isolates was extracted and *recA* and draft whole genome sequences were generated as described in a previous publication [19]. Multilocus sequence analysis was performed as described earlier [19] except that gene fragments were obtained from the draft genomes. Gene number assignment to each unique allele and assignment of sequence type to a unique allelic profile were done using the BCC pubMLST database [20]. All sequences are publicly available at http://pubmlst.org/bcc/.

Antibiotic susceptibility testing was performed to avibactam-ceftazidim, amikacin, cefotaxime, ceftazidime, ceftolozane-tazobactam, cefuroxime, ciprofloxacin, gentamicin, imipenem, meropenem, piperacillin/tazobactam, tobramycin and trimethoprim-sulfametoxazole. The minimum inhibitory concentration (MIC) was determined by ISO broth microdilution method (Sensititre, TREK Diagnostics/Thermo Fischer Scientific), and gradient strip method (Liofilchem s.r.l, Roseto degli Abruzzi).

According to the European Committee on Antimicrobial Susceptibility Testing (EUCAST), establishment of MIC breakpoints for categorizing a BCC strain as sensitive, intermediate or resistant is currently not possible. Therefore, the PK-PD (Pharmacokinetic-Pharmacodynamic, i.e. non-species related) breakpoint from EUCAST/NordicAST 2019 was used for interpretation of the possible sensitivity of the antimicrobial agents tested.

The Regional Resource Center of Infection Control at UNN conducted a systematic review of the infection control and decontamination (i.e. cleaning, disinfection and sterilization) routines during two site visits at the private outpatient clinic. Samples from liquid drugs and products, medical devices, IOLs and multiple environmental sites, including repeated samples from all sink drains, were cultured in the laboratory.

## Results

*B. contaminans* endophthalmitis was confirmed in six cases among 290 patients (2.1%) who underwent cataract surgery in a private outpatient clinic between January 4th to 28th 2019. The *rec*A sequences of all six isolates were 100% identical and identified as *B. contaminans* through comparison with the PubMLST database*.* All isolates had an identical allelic profile (*atpD*-64, *gltB*-80, *gyrB*-76, *recA*-89, *lepA*-105, *phaC*-97, *trpB*-70) and belong to the outbreak strain ST-102. Although the genotyping analysis indicated a common source of infection, no source was identified by the The Regional Resource Center of Infection Control at UNN.

**Fig. 1:**
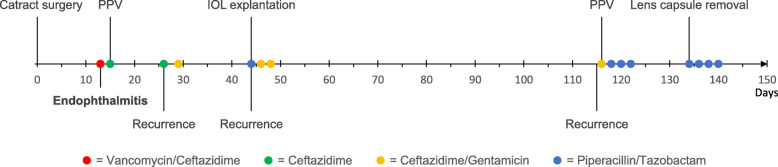
Timeline of important clinical events and treatments in Case 1 from days 0 to 150 after cataract surgery. The colored dots indicate intravitreal antibiotic injections. PPV: Pars plana vitrectomy. IOL: Intraocular lens

The first case (case 1) is described in detail to illustrate the complex disease course and highlight important clinical events and is summarized in Fig. 1. The patient underwent an uneventful cataract surgery on the left eye 13th of January 2019 (day 0) and presented on day 13 after surgery with VA hand motion and severe intraocular inflammation in the left eye. A vitreous tap and injection of vancomycin, ceftazidime and dexamethasone were performed at admission. *B. contaminans* was confirmed in the vitreous sample. On day 15, the patient underwent full PPV, a vitreous tap and injection of ceftazidime, followed by a clinically favorable response, except from elevated intraocular pressure (IOP) treated with topical medication. On day 24, VA had improved to 0.8. However, the patient experienced an acute recurrence with VA hand motion, IOP 40 mmHg and severe inflammation on day 26. A vitreous tap and injection of ceftazidime and dexamethasone were performed. Furthermore, an additional vitreous injection with ceftazidime and gentamicin was performed on day 29. In addition, oral ciprofloxacin 750 mg was administrated twice daily for 25 days. The vitreous sample confirmed persistent *B. contaminans* infection. Once more, the clinical response seemed favorable. However, the case presented on day 44 with an acute exacerbation with VA hand motion and severe inflammation. The IOL was explanted and a thorough PPV including intravitreal injection of piperacillin/tazobactam was performed. A complete removal of the lens capsule was not possible due to impaired visibility during the surgery. Peroperatively, retinal ischemia and inflammatory membranes were found. *B. contaminans* was confirmed in the vitreous tap and from the IOL. Vitreous injections of gentamicin and ceftazidime were repeated on days 46 and 48.

The patient gradually recovered to VA 0.8, but presented with an acute exacerbation on day 115. A vitreous tap and intravitreal injection of ceftazidime and gentamicin were performed on day 116. *B. contaminans* was again confirmed from the vitreous tap and intravitreal injections of piperacillin/tazobactam were repeated on days 118, 120, and 122. It was decided to perform a total lens capsule removal combined with piperacillin/tazobactam injection on day 134, followed by three additional injections of piperacillin/tazobactam (days 136, 138 and 140). *B. contaminans* was confirmed from the explanted lens capsule remnants, a total of 134 days after the initial cataract surgery. The patient received a total of 15 intravitreal injections of antibiotics. After the removal of the lens capsule, the patient had no recurrences. A secondary IOL was implanted on day 288, resulting in a good visual outcome.

The demographics, clinical course and treatment of all cases with confirmed *B. contaminans* endophthalmitis are summarized in Table 1. Case 3 was admitted with suspected postoperative endophthalmitis in the left eye. However, the following day, VA had dramatically declined to hand motion and severe inflammation was also found in the right eye. The patient underwent bilateral PPV, vitreous tap and intravitreal injection of ceftazidime and gentamicin. *B. contaminans* was confirmed in the left eye only, and the right eye recovered without any additional treatment. Case 5 did not have recurrent endophthalmitis symptoms. However, it was decided to explant the IOL and lens capsule based on the experience from the other cases. *B. contaminans* was confirmed from the explanted IOL and lens capsule.

**Table 1:** Demographics, clinical course and treatment in cases with *Burkholderia contaminans* endophthalmitis

	Case 1	Case 2	Case 3	Case 4	Case 5	Case 6
Sex / age (years)	Male / 73	Male / 89	Male / 74	Male / 86	Female / 59	Male / 72
Date of surgery	13.01.19	14.01.19	13.01.19	13.01.19	13.01.19	08.01.19
Eye(s) operated	Left	Both	Both	Left	Both	Both
Eye with endophthalmitis	Left	Left	Left	Left	Right	Left
Date of diagnosis	26.01.19	26.01.19	28.01.19	29.01.19	18.02.19	30.04.19
Time to diagnosis (days)	13	12	15	16	36	112
Time to recurrence (days)	13 18 71	16	13 16	27	–	32 43
Number of recurrences	3	1	2	1	0	2
Intravitreal antibiotics (n)	Vancomycin (1) Ceftazidime (7) Piperacillin /tazobactam (8) Gentamicin (4)	Vancomycin (1) Ceftazidime (7) Piperacillin/ tazobactam (3) Gentamicin (4)	Ceftazidime (5) Gentamicin (5)	Ceftazidime (2) Piperacillin/ tazobactam (3) Gentamicin (2)	Ceftazidime (3) Piperacillin/ tazobactam (4) Gentamicin (3)	Vancomycin (3) Ceftazidime (3) Piperacillin/ tazobactam (2)
Systemic antibiotics	Ciprofloxacin 750 mg × 2	Ciprofloxacin 500 mg × 2	Ciprofloxacin 750 mg × 2	Ciprofloxacin 250 mg × 2	Ciprofloxacin 500 mg × 2	–
Date of IOL/ lens capsule removal	26.02.19 /27.05.19	28.02.19	–	01.03.19	05.03.19	18.07.19
Number of treatments	15	10	5	5	7	5
Date of secondary IOL surgery	28.10.19	21.08.19	–	14.10.19	17.06.20	21.10.19
Time to secondary IOL (days)	288	219	–	274	521	286
Final CDVA	1.2	0.9	1.0	1.0	0.8	0.8

*CDVA* (corrected distance visual acuity measured by Snellen chart). *IOL* (intraocular lens). Cases are presented according to date of hospital admission

Case 6 presented with moderately increased anterior chamber inflammation in the left eye 1 month after the cataract surgery. Because there was no vitreous infiltration, endophthalmitis was not suspected and the case was treated with topical steroids with a seemingly favorable response. However, 112 days after the cataract surgery, the case presented with an acute exacerbation with severe anterior chamber inflammation and vitreous infiltration. PPV and injection of ceftazidime and vancomycin were performed with a seemingly favorable clinical response. However, endophthalmitis symptoms recurred 1 month later, and a vitreous tap and injection of ceftazidime and vancomycin were repeated. There was no growth of bacteria from the samples from the PPV or vitreous tap. Six weeks later, the case presented with a second recurrence and the IOL and lens capsule were explanted. Ceftazidime and vancomycin were injected peroperatively followed by two intravitreal injections of piperacilin/tazobactam 2 days apart. *B. contaminans* was confirmed from the explanted IOL and lens capsule. The case had no further recurrences and later received a secondary IOL.

The mean time from surgery to admission with suspected endophthalmitis was 34 days (range 12–112 days). All cases had a similar clinical presentation including VA hand motion, and rapid progressing and extensive anterior chamber and vitreous inflammation. The cases had a seemingly favorable response to the intravitreal antibiotics given with improved VA and diminishing inflammation. However, acute recurrences occurred after long time periods. The cases experienced between 0 and 3 recurrences (mean 1.5), which occurred between 12 and 71 days (mean 28 days).

Due to persistent infection, the cases received between 5 and 15 treatments (i.e. intravitreal injection of antibiotics, IOL and lens capsule explantation and PPV, mean 7.8). In total, five of the six IOLs and lens capsules were explanted. *B. contaminans* was detected in all explanted lens capsules. The IOL in case 3 was not explanted due to the patient’s wish and favorable clinical response to repeated intravitreal antibiotics and PPV. All patients were treated with intravitreal injection of ceftazidime. Five additionally received piperacillin/tazobactam. Moreover, five cases were treated with oral ciprofloxacin. Secondary IOLs were implanted in five cases between 219 and 521 days after the initial cataract surgery. The final corrected distance visual acuity (CDVA) was between 0.8 and 1.2, and all cases had final CDVA ≥0.8.

The MIC-values (mg/L) for the different antimicrobial agents tested are presented in Table 2.

**Table 2:** Minimum inhibitory concentration (MIC) values for *Burkholderia contaminans*

Antibiotic	MIC-values (mg/ml)
Amikacin	< 2
Cefotaxime	> 32
Ceftazidime	4
Cefuroxime	64
Ciprofloxacin	0.5
Colistin	> 128
Gentamicin	2
Imipenem	4
Meropenem	4
Piperacillin/tazobactam	2
Tobramycin	1
Trimethoprim-sulfametoxazol	0.5

## Discussion

In the present study, we report the clinical course, treatment and visual outcome of a *B. contaminans* endophthalmitis outbreak following cataract surgery treated with repeated intravitreal antibiotic injections, PPV, and IOL and lens capsule removal, resulting in a good visual outcome. The treatment approach was initially based on standard recommendations for postoperative endophthalmitis, i.e. a vitreous tap and intravitreal injection of antibiotics in two cases and primary PPV and intravitreal injection of antibiotics in four cases. The first two cases of the present outbreak were admitted on the same day and both received intravitreal injection of vancomycin and ceftazidime according to the ESCRS guidelines [17]. *B. contaminans* is a Gram-negative bacterium and therefore intrinsically resistant to vancomycin. Based on the MIC values, the use of ceftazidime, piperacillin/tazobactam, ciprofloxacin and gentamicin could be recommended, whereas cefuroxime could not be recommended. Due to recurrent endophthalmitis, the treatment was extended to include repeated intravitreal injections of antibiotics according to the MIC values for all cases except case 6. The long delay between surgery and endophthalmitis symptoms (112 days) in case 6 was believed to indicate an alternative causative bacteria, such as *Cutibacterium acnes* (formerly *Propionibacterium acnes*), known to cause chronic late onset endophthalmitis [21]. Therefore, the patient received three injections of vancomycin and ceftazidime before *B. contaminans* was confirmed and MIC values obtained.

Despite of PPV and repeated intravitreal injections of recommended antibiotics including ceftazidime, gentamicin and piperacillin/tazobactam, the infection persisted in all except one case. It was therefore decided to explant the IOLs including the lens capsule, which eradicated the infection in all cases except from case 1 who had residual lens capsule remnants. As a result, the lens capsule remnants were removed, and as hypothesized, *B. contaminans* was confirmed from the lens capsule remnants and the infection was subsequently eradicated. This suggests that intravitreal antibiotic injection and removal of both the IOL and the entire lens capsule were required in order to eradicate the infection in most cases.

There was a large variability in time to diagnosis ranging from 12 to 112 days. Nonetheless, five of the cases were diagnosed with endophthalmitis within 6 weeks following surgery and hence defined as acute postoperative endophthalmitis according to the ESCRS guidelines [17]. The last case (case 6) may be defined as chronic or delayed onset endophthalmitis, although in retrospect, moderately increased anterior chamber flare was detected within 4 weeks of surgery. Similarly, the time interval from treatment to recurrent symptoms varied from 13 to 71 days. Regardless of the large variability in time to diagnosis and recurrent symptoms after treatment, the clinical presentation was characterized by rapidly progressive and severe ocular inflammation in all cases.

Persistent endophthalmitis after intravitreal antibiotic injection may be caused by drug resistance. A lack of binding sites on the lipopolysaccharides of BCC bacteria has been reported to cause intrinsic resistance to polymyxins and aminoglycosides, although MIC values from the current *B. contaminans* isolates indicated sensitivity for aminoglycosides [22]. BCC bacteria may also be resistant to β-lactams due to a combination of outer membrane impermeability and inducible chromosomal β-lactamases [23, 24]. Moreover, at least one efflux pump system has been described that confers intrinsic resistance to tetracycline, chloramphenicol and ciprofloxacin [25]. Due to these resistance mechanisms, multiple drug resistance is common in BCC. In one study, 50% of BCC isolates were resistant in vitro to all 10 commonly used agents tested [7]. In addition, BCC has a capacity for rapid mutation and adaptation to demanding environments [8]. The present MIC values were stable throughout the entire outbreak, indicating that *B. contaminans* was susceptible to the antibiotics used. Nonetheless, the infection persisted despite of repeated antibiotic injections in five of six cases. In addition, *B. contaminans* was detected from all explanted lens capsules, including the lens capsule remnants in case 1 in which the IOL had been explanted 3 months earlier, whereas no case experienced persistent infection after complete lens capsule removal. All these observations indicate that *B. contaminans* has the ability to colonize the lens capsule and resist antibiotic treatment, possibly by biofilm formation. This is in accordance to a previous publication showing that BCC has the ability to form biofilms in order to increase antibiotic resistance [26].

Intravitreal injection of antibiotics is the mainstay treatment of postoperative endophthalmitis. However, according to ESCRS guidelines [17], adjunctive systemic antibiotics may be recommended in some cases. Based on the MIC values suggesting an antimicrobial effect of ciprofloxacin and reports suggesting vitreous penetration of systemic ciprofloxacin [27], five cases were treated with oral ciprofloxacin although our data could not support any additional effect of oral antibiotics. Furthermore, case 1 received eight intravitreal injections of piperazillin/tazobactam due to multiple recurrences, which appeared to be well tolerated.

*B. contaminans* is one of the most commonly reported contaminants of pharmaceutical products [8]. The current *B. contaminans* outbreak strain, ST-102, has been involved in several earlier outbreaks related to contaminated pharmaceutical products worldwide, including an outbreak in a haemodialysis unit [28], a nasal spray outbreak [29] and more recently an outbreak due to contaminated liquid docusate sodium [30]. Although BCC contamination of trypan blue dye, local anesthetic eye drops and silicon oil has been identified in outbreaks of endophthalmitis, the underlying source of infection is unknown in most outbreaks of postoperative BCC endophthalmitis, which agrees with our results [11–16]. The outpatient clinic conducted an independent investigation to identify the source of infections. However, the results have not been made available to the authors for publication.

## Conclusions

Based on the experience from the present endophthalmitis outbreak with persistent infection and detection of *B. contaminans* in the explanted lens capsules after repeated intravitreal antibiotic injections and PPV, we postulate that *B. contaminans* has the ability to colonize the lens capsule and resist antibiotic treatment, possibly by biofilm formation. Although one patient with confirmed *B. contaminans* endophthalmitis had a favorable outcome without IOL and lens capsule explantation, we recommend total lens capsule and IOL explantation for *B. contaminans* endophthalmitis following cataract surgery, at least if the first line vitreous tap or PPV and intravitreal antibiotic injection fail. Furthermore, based on experiences from the current *B. contaminans* outbreak, we recommend a persistent and intensive treatment approach, which in our experience may lead to a favorable visual result.

## Data Availability

The data that support the findings of this study are based on patient journal review and not publicly available.

## Abbreviations

BCC: *Burkholderia cepacia* complex
CDVA: Corrected distance visual acuity
ESCRS: European Society of Cataract & Refractive Surgeons
EUCAST: European Committee on Antimicrobial Susceptibility Testing
IOL: Intraocular lens
IOP: Intraocular pressure
MALDI-TOF: Matrix Assisted Laser Desorption Ionization Time-Of-Flight mass spectrometry
MIC: Minimum inhibitory concentration
PK-PD: Pharmacokinetic-Pharmacodynamic
PPV: Pars plana vitrectomy
UNN: University Hospital of North Norway
VA: Visual acuity
